# Simultaneous Determination of Reducing Sugars in Honey by Capillary Zone Electrophoresis with LIF Detection Using Low-Toxicity 2-Picoline Borane and APTS for Pre-Capillary Derivatization

**DOI:** 10.3390/ijms26157569

**Published:** 2025-08-05

**Authors:** Joanna Bulesowska, Michał Pieckowski, Piotr Kowalski, Tomasz Bączek, Ilona Olędzka

**Affiliations:** 1 Department of Pharmaceutical Chemistry, Medical University of Gdańsk, 80-416 Gdańsk, Poland; gd52082@gumed.edu.pl (J.B.); mpiec@gumed.edu.pl (M.P.); piotr.kowalski@gumed.edu.pl (P.K.);; 2 Department of Nursing and Medical Rescue, Institute of Health Sciences, Pomeranian University in Słupsk, 76-200 Słupsk, Poland

**Keywords:** 2-picoline borane, APTS, capillary zone electrophoresis, fructose, glucose, honey, LIF detection, maltose, mannose, sugars derivatives

## Abstract

This study aimed to develop a reliable method for profiling reducing sugars in honey using capillary zone electrophoresis with laser-induced fluorescence detection (CZE-LIF). Reducing sugars were derivatized with 8-aminopyrene-1,3,6-trisulfonic acid (APTS) in the presence of 2-picoline borane, a safer alternative to sodium cyanoborohydride. Key parameters influencing the derivatization efficiency—temperature, pH, incubation time, and reagent concentrations—were systematically optimized. The highest labeling efficiency for glucose, mannose, and maltose was achieved at 50 °C in 0.5 M citric acid with 0.1 M APTS, while fructose showed low reactivity due to its ketose structure. To reduce the background signal from excess reagents, three cleanup strategies were evaluated. Liquid–liquid extraction with ethyl acetate effectively removed unreacted APTS without significant analyte loss, whereas solid-phase extraction and microextraction caused substantial losses of hydrophilic sugars. The method showed good linearity (0.5–10 mM, R^2^ > 0.994), precision (RSD 0.81–13.73%), and accuracy (recoveries 93.47–119.75%). Stability studies indicated that sugar standards should be stored at –20 °C. The method was successfully applied to the analysis of four nectar honeys—rapeseed, acacia, phacelia, and dandelion—revealing differences in glucose and fructose content related to botanical origin. The results confirm the suitability of CZE-LIF for sensitive and selective carbohydrate analyses in complex food matrices.

## 1. Introduction

It is estimated that there are approximately 300–400 varieties of honey distributed globally, and their availability is determined by factors such as the region in which nectar-producing plants grow. The existing species diversity leads to differences not only in taste, aroma, consistency, and color, but also in the properties of individual honeys due to variations in their composition. As a result, honey is a popular bee product not only as a sweet addition to meals, but also due to its health-promoting properties. These include antibacterial and antiseptic effects, support for wound healing, improved digestion, immune system enhancement, and relief of coughs and sore throats. The sugars present in honeys are of primary importance in determining their nutritional value. Therefore, to confirm the authenticity and quality of these products, analyses are performed using various methods targeting sugar compounds [1,2,3].

Spectroscopic methods (IR, NMR, and fluorescence spectrophotometry) are characterized by simple sample preparation, low consumption of organic solvents and other reagents, and the ability to analyze intact food samples in a non-invasive and time-efficient manner. However, these techniques also have several limitations, including low selectivity, the inability to analyze compound mixtures, and a strong influence of contaminants on the shape of the spectrum obtained [4]. Chromatographic methods (LC, HPLC, and GC) allow high-level separation and quantification of micro- and macronutrients with similar structures present in complex matrices, such as food products. However, these techniques are costly, time-consuming, and require relatively large volumes of organic solvents and samples [1,4,5,6]. Electrochemical methods (voltammetry, electronic tongue, and CE) are simple, inexpensive, and time-efficient techniques that provide information about the redox properties of individual honey components. These features are particularly useful in detecting adulterants such as glucose or sucrose syrups. Nevertheless, these methods may suffer from lower reproducibility of migration times and reduced sensitivity, especially when compared to HPLC [1,4].

In the present study, a variant of capillary electrophoresis (CE)—capillary zone electrophoresis (CZE)—was applied as the separation method. CE is a technique that enables the separation of compounds present in attomole concentrations under the influence of a high voltage applied across the capillary. Capillary electrophoresis is widely applied in chemical and biological analysis, including pharmaceutical research, environmental sample characterization, and the separation and identification of chemical compounds, including optical isomers [7]. CE can also be used to analyze the composition of food products, such as honey and syrups, as it allows for the separation of both inorganic and inorganic compounds, including carbohydrates, which are the main constituents of these products. In sugar analysis, CE offers the following several advantages: short analysis time, high efficiency, low consumption of reagents (reductants, organic acids, and labeling agents), minimal waste generation, and the possibility of working with small sample volumes (50 μL) [8,9,10,11,12]. To separate and determine the analytes, a capillary zone electrophoresis system equipped with a laser-induced fluorescence (LIF) detector was used. LIF detection is one of the most sensitive and specific detection methods in CE. Compared to other optical methods, it offers significantly better detection parameters, as CE provides favorable conditions (small sample volumes [nL] and short optical path lengths determined by the capillary inner diameter [25–75 μm]). Moreover, LIF exhibits a higher detection efficiency at a given wavelength compared to UV lamps [13]. The analysis of carbohydrates present in honey required the use of appropriate reducing and labeling agents [1,3,14]. 2-Picoline borane was selected as the reducing agent due to its higher stability and lower selectivity toward solvents in comparison to sodium cyanoborohydride (NaCNBH_3_), as well as its lower toxicity (it helps avoid the formation of toxic byproducts, such as HCN and NaCN) [5,15,16,17]. 8-Aminopyrene-1,3,6-trisulfonic acid trisodium salt (APTS) was chosen as the fluorophore due to its ability to absorb electromagnetic radiation and covalently bind, via its free amino group, to the reduced sugars. This enables their detection using CZE with LIF detection. According to the literature reports, sodium cyanoborohydride (NaBH_3_CN) and 2-picoline borane are the most frequently employed reducing agents in the reductive amination of sugars [5,17,18]. In recent years, a marked trend has emerged toward replacing NaBH_3_CN with safer and more environmentally benign alternatives, particularly 2-picoline borane, without compromising derivatization efficiency [6,15]. The selection of an appropriate reducing agent is primarily governed by specific analytical requirements and its compatibility with the chosen fluorophore.

The aim of this study was to develop an optimal method for the determination of selected sugars, including glucose, fructose, mannose, and maltose, in honey samples using capillary zone electrophoresis (CZE) coupled with laser-induced fluorescence (LIF) detection. Additionally, another objective was to establish an optimized procedure encompassing the reduction and labeling of the target sugars for their determination in the aforementioned samples. The influence of labeling reagents, such as APTS, and the reducing agent 2-picoline borane, as an alternative to the commonly used but highly toxic NaBH_3_CN, was evaluated. Experimental conditions affecting the efficiency of the derivatization reaction, including reagent concentrations, solvent type, pH, incubation time, and reaction temperature, were optimized. A key contribution of this work is the comparative assessment of sample cleanup strategies (LLE, SPE, and SPME) to remove excess derivatization reagents. The findings highlight the potential of producing safer 2-picoline borane compared to NaBH_3_CN and the SPE-based cleanup of honey samples as tools in routine carbohydrate analysis workflows.

## 2. Results and Discussion

### 2.1. Optimization of Reaction Conditions for Sugar Derivatization

In our study, 2-picoline borane was employed as the reducing agent to enhance both the safety of the derivatization procedure and the efficiency of the reductive amination reaction. Compared to sodium cyanoborohydride (NaCNBH_3_), which has been widely used in similar applications, 2-picoline borane offers significant advantages in terms of laboratory safety. Importantly, 2-picoline borane does not generate toxic byproducts, such as hydrogen cyanide (HCN), which are a significant concern in the use of NaCNBH_3_, especially under acidic conditions. In addition to its safer handling profile, the literature data suggest that 2-picoline borane can achieve comparable or slightly higher derivatization yields for several monosaccharides and oligosaccharides under optimized conditions [5]. Similarly, the aim of the study presented by Křenkova et al. was to apply CE-LIF to the analysis of reducing sugars or oligosaccharides, using 2-picoline borate as a safer reducing agent in the derivatization process and to compare APTS as a commonly used fluorescent label with the new fluorescent label 4,4′,4″(8-aminopyrene-1,3,6-trisulfonyl)tris(1-methylpiperazine) (APTMP) [18]. Therefore, 2-picoline borane represents a valuable alternative for carbohydrate labeling, especially in routine analytical protocols where both analytical performance and operator safety are paramount. In our method, the use of 2-picoline borane supported the efficient and reproducible labeling of target sugars, aligning with the reported data on its efficacy [5,15,17].

The selection of an optimal incubation temperature and time during the labeling of sugars with APTS and 2-picoline borane as the reducing agent is critical for achieving high derivatization efficiency and sensitivity in CZE-LIF analysis. Labeling of sugars with APTS involves the formation of a Schiff base between the amino group of APTS and the aldehyde group of the reducing sugar, which is subsequently reduced to a stable amine derivative by 2-picoline borane. This reaction requires carefully controlled conditions of pH, temperature, and incubation time to proceed effectively. Insufficient temperature (<30 °C) may lead to incomplete condensation and reduction, resulting in weak LIF signal intensity due to poor labeling efficiency. The literature reports suggest that an incubation temperature in the range of 35–55 °C provides optimal conditions for effective labeling [9,19]. As reported in the literature data, glucose, maltose, and mannose exhibit optimal labeling efficiency at temperatures in the range of 45–55 °C, whereas fructose, being a ketose, may require slightly higher temperatures (~55 °C) to achieve full conversion to its reactive cyclic form [9,18,19,20]. However, elevated temperatures above 60 °C may lead to the degradation of APTS or the formation of undesirable byproducts resulting from thermal decomposition of the target sugars, particularly in the case of fructose. The optimal incubation time is determined by the kinetics of the condensation and reduction reactions, which typically occur over a period of 1 to 4 h, depending on the temperature. Incubation for 2 h at 50–60 °C is frequently reported as effective for the majority of reducing sugars. Although extending the incubation time beyond 4 h may slightly improve derivatization yield, it also increases the risk of background signal and byproduct formation. For fructose, which exhibits lower reactivity due to its structural characteristics, longer incubation times (3–4 h) have been found to enhance labeling efficiency [21]. Based on our observations, glucose, mannose, and maltose are most effectively labeled at 50 °C. Among the temperature conditions tested (ranging from 30 to 75 °C), 50 °C was found to be optimal for the derivatization of these three sugars. In the study conducted by Fang et al., optimal derivatization efficiency for several saccharides was achieved under incubation conditions of 40 °C for 3 h. Interestingly, at higher temperatures (50 and 60 °C), a slight decrease in reaction efficiency was observed over time [5].

In our experiment concerning fructose, the most effective derivatization effect was achieved at 50 °C for 4 h (see Figures S1–S4). At 30 °C, the reaction yielded slightly lower efficiency, while at 40 °C—the intermediate temperature—the lowest signal intensity was observed, which may seem counterintuitive. However, such anomalies can be explained by the chemical nature of fructose, which is prone to transformations including isomerization, degradation, and condensation. At 40 °C, unstable intermediates may form and decompose more rapidly than at 30 °C, whereas at 50 °C, the reaction likely proceeds faster and stabilizes the products before degradation occurs. Additionally, side reactions, such as aldol condensation or acid/base-catalyzed degradation, could be more prominent at intermediate temperatures, while at 50 °C they may be outcompeted by the main derivatization pathway. Kinetic and thermodynamic factors also play a role. At 30 °C, the reaction may proceed slowly but yield more stable products, whereas at 50 °C, higher energy input accelerates the reaction and favors thermodynamically stable derivatives. At 40 °C, the reaction may be too slow to stabilize products, yet too fast for proper kinetic control. The literature data also highlight that fructose consistently shows lower derivatization efficiency in sugar mixtures. This is due to its slower reaction initiation, which allows more reactive sugars, like glucose and mannose, to outcompete it for APTS and the reducing agent, resulting in diminished or undetectable fructose signals during short incubation periods [18,21,22,23].

The next important factor is the pH value of the reaction medium, which plays a critical role in the labeling process involving APTS and 2-picoline borane, as it directly affects the protonation state of the amino group in APTS, which must remain unprotonated to effectively react with the aldehyde group of reducing sugars. Maintaining an optimal pH is essential to ensure the stability of the intermediate Schiff base, as studies have shown that overly acidic conditions can destabilize this intermediate [24]. Furthermore, pH also influences the efficiency of the reduction step catalyzed by 2-picoline borane, which performs best in mildly acidic conditions. According to the literature data, a pH of approximately 4.5 is most commonly employed, as it offers a compromise between reaction rate and the stability of the resulting derivatives [24,25,26,27]. Within this pH range, APTS remains sufficiently reactive, the aldehyde group of the sugar (in its open-chain form) is accessible, and the reduction of the Schiff base by 2-picoline borane proceeds efficiently. Moreover, the findings reported by Evangelista et al. indicate that the highest stability of sugar–APTS adducts is achieved in the presence of citric acid (pKa 3.13). In contrast, adducts formed in the presence of acetic acid (pKa 4.75) exhibited lower stability, suggesting that the choice of buffer not only influences reaction kinetics, but also impacts the structural integrity of the final derivatives [24,25,26,27]. In our study, 0.5 M citric acid (pH 1.7) was used to ensure appropriate pH conditions during the derivatization reaction. Based on preliminary optimization using mannose as a model compound, 0.5 M citric acid was found to be the optimal concentration for achieving clear electropherogram signals. At its lower concentrations, only a flat baseline was observed, with no detectable analyte peaks. Additionally, we observed that for fructose, the use of 0.5 M citric acid slightly improved the reaction efficiency. These findings support the use of higher citric acid concentrations to enhance labeling efficiency and signal clarity, particularly for sugars with lower reactivity.

The effect of APTS concentration on signal intensity was evaluated in the range of 0.05–0.2 M. A final concentration of 0.1 M APTS was selected, as lower concentrations (<0.1 M) yielded satisfactory labeling efficiency for individual sugars, but resulted in significantly weaker signals when analyzing mixtures. Conversely, at higher concentrations (>0.1 M), excessive background signals from APTS interfered with analyte detection due to peak overlapping. The literature data indicate that commonly used APTS concentrations fall within the 20–50 mM range. For instance, Varadi et al. reported that an optimal concentration of 40 mM APTS (in 20% acetic acid), incubated for 2 h at 37 °C, provided efficient reductive amination [16,21]. They also noted that while overnight incubation led to the highest signal intensity, it caused significant degradation of sialylated sugar structures.

It should be noted that, in the present study, challenges were also encountered in the labeling and detection of fructose. The lower efficiency of derivatization with APTS and 2-picoline borane, compared to other sugars, may be attributed to several factors. Firstly, fructose is a ketose, possessing a ketone group instead of an aldehyde, as found in aldoses like mannose. Ketone groups are generally less reactive in reductive amination reactions, which may hinder the efficient interaction with APTS and the reducing agent. Additionally, in solution, fructose exists as a mixture of tautomers—cyclic (furanose and pyranose) and linear forms. Since only the linear form participates in the derivatization reaction, the equilibrium between these forms can limit the overall reaction yield. Fructose is also more prone to isomerization and degradation, particularly under elevated temperatures or in alkaline conditions, both of which are commonly used to activate the reaction with APTS. The literature reports confirm that ketose sugars may require modified reaction parameters to achieve efficient labeling, as conditions optimal for typical reducing sugars (aldoses) are often insufficient [22,26].

**Figure 1:**
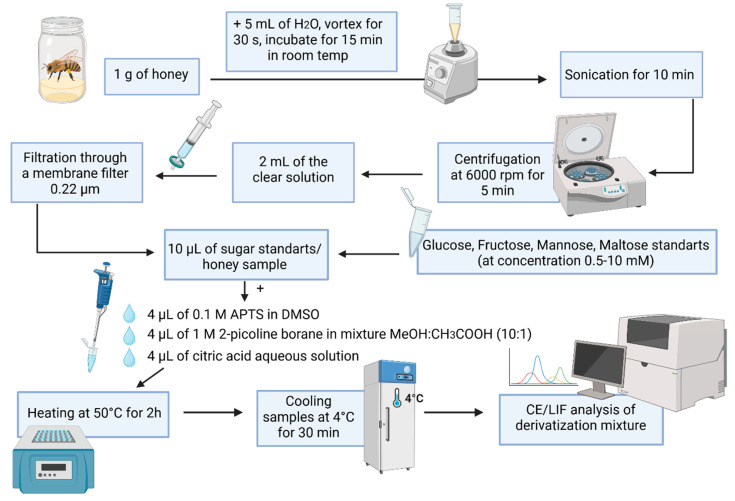
Schematic representation of the labeling procedure for samples containing the analyzed sugars or honey samples using APTS and 2-picoline (created with BioRender, Joanna Bulesowska, 30 June 2025 [https://www.biorender.com]/).

Taking all these dependencies into account, we developed a protocol enabling the efficient derivatization of three sugars—glucose, mannose, and maltose—using the safer reducing agent 2-picoline borane. The procedural scheme is presented in Figure 1. However, under these conditions, the derivatization of fructose proved to be ineffective, requiring a modified protocol. Consequently, a simultaneous analysis of all four sugars is not feasible.

### 2.2. Assessment of Methods for Removing Excess Derivatization Reagents

The analyses revealed the presence of interferences originating from the derivatization reagents. To improve the resolution of the method, efforts were made to remove excess reagents from the samples. The removal of unreacted APTS and 2-picoline borane following analyte labeling is critical for enhancing the performance of CZE-LIF analysis. Residual derivatization agents can interfere with fluorescence detection or negatively affect electrophoretic separation. To assess the impact of reagent removal, several cleanup approaches were tested and evaluated, including liquid–liquid extraction (LLE), solid-phase extraction (SPE), and solid-phase microextraction (SPME).

#### 2.2.1. A Liquid–Liquid Extraction (LLE)-Based Approach

**Figure 2:**
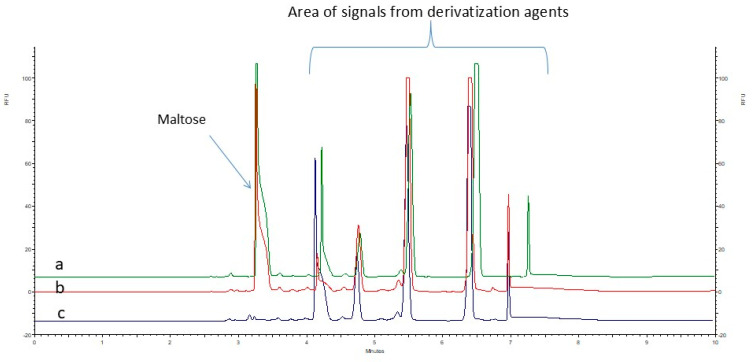
Electropherograms of a sample containing maltose at a concentration of 10 mM before and after purification using the LLE method: (a) sample before LLE purification, (b) sample after LLE purification, and (c) blank sample (without analytes) after LLE purification.

To remove excess unbound APTS, a sample cleanup procedure based on LLE was tested. Ethyl acetate was selected as the extraction solvent in a 2:1 ratio (ethyl acetate:sample) due to its lower polarity compared to water, lower density—which facilitates phase separation—and its reduced tendency to cause interferences in CZE-LIF analysis compared to more commonly used solvents such as dichloromethane. Due to the presence of sulfonic acid groups, APTS–sugar conjugates are highly hydrophilic and remain preferentially in the aqueous (lower) phase. In contrast, unbound APTS, being less polar, is more soluble in the organic phase and is effectively extracted into the ethyl acetate layer. The electrophoretic profiles of the sample containing the model sugar, maltose, after APTS labeling in the presence of 2-picoline borane, showed no significant differences before and after the application of liquid–liquid extraction (LLE) as a purification step. This indicates that the LLE procedure is not effective in removing excess labeling reagents from the sample as is presented in Figure 2.

#### 2.2.2. A Solid-Phase Extraction (SPE)-Based Approach

A SPE procedure using C18 columns was also tested for sample cleanup efficiency. The C18 sorbent, composed of hydrophobic octyl chains, selectively retains non-polar compounds through van der Waals interactions, with electrostatic forces playing a minimal role. In aqueous matrices, hydrophobic analytes tend to interact more strongly with the non-polar C18 surface.

Unlike LLE, where solubility of analytes in extraction solvent governs on separation efficiency, in SPE retention depends primarily on their hydrophobicity. While APTS–sugar conjugates are largely hydrophilic due to sulfonic and hydroxyl groups, the aromatic ring in APTS molecule allows for partial retention on the C18 sorbent. Free APTS, being more hydrophilic and lacking significant hydrophobic interactions, does not bind in aqueous conditions. The proposed method efficiently removes unbound APTS, 2-picoline borane, and organic residues (e.g., ACN) without requiring sample evaporation.

**Figure 3:**
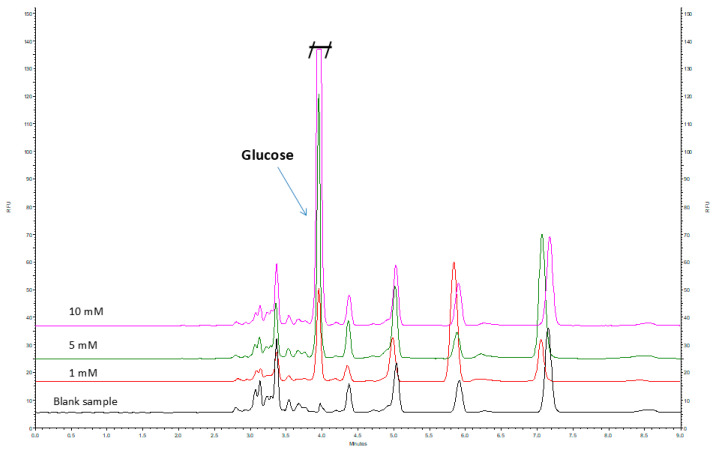
Electropherograms of samples containing glucose at concentrations of 1, 5, and 10 mM, as well as a blank sample, after purification using the SPE C18 procedure.

The application of SPE prior to analysis effectively removed excess reagents used during the reductive amination process, as demonstrated by the comparison of two blank samples, one of which underwent the cleanup procedure (Figure S5). The electropherograms clearly show significantly reduced signals corresponding to excess APTS in the sample subjected to the SPE procedure. However, with respect to the analyte signals, it was observed that the applied SPE method resulted in a slightl decrease in the signal intensity of the investigated sugars (Figure 3 and Figure S6).

#### 2.2.3. A Solid-Phase Microextraction (SPME)-Based Approach

The final technique tested was the adaptation of the SPE C18 cleanup procedure to solid-phase microextraction (SPME), which required a consideration of key differences between the methods [28]. Both SPE and SPME with C18 sorbents rely on hydrophobic interactions. SPE columns, with greater sorbent mass and surface area, enable efficient retention from larger sample volumes. SPME employs a sorbent-coated fiber with a lower capacity, but allows faster equilibrium. In SPE, near-complete extraction is possible as the entire sample passes through the column. In contrast, SPME depends on analyte adsorption from the matrix, with efficiency influenced by the contact time and surface area. Optimization of extraction conditions (typically 10–30 min with agitation) is essential. Due to the limited sorbent capacity, sample dilution is often required to reduce matrix effects. SPME minimizes solvent use and supports direct integration with analytical systems, such as CE. However, its limitations include its lower capacity, need for precise optimization, and potentially reduced recovery for highly hydrophilic compounds.

**Figure 4:**
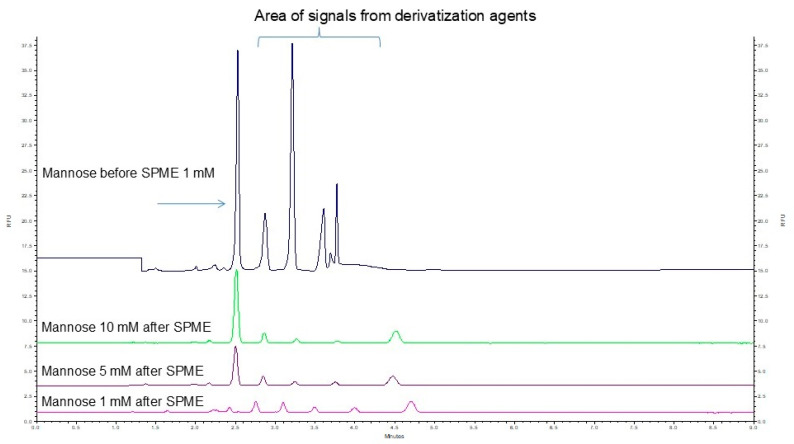
Electropherograms of samples containing mannose before and after purification using the SPME C18 procedure.

In this study, SPME fibers coated with a C18 sorbent were used. Prior to extraction, the fibers were conditioned for 30 min in 1.5 mL of an acetonitrile–water solution (50:50, *v*/*v*) and then rinsed in deionized water for 10 s. Pre-prepared samples (see Section 3.4.1 and Figure 1) were placed in a 96-well plate and extracted for 30 min under gentle agitation according to the SPME procedure described in Section “A Solid-Phase Microextraction (SPME)-Based Approach”. After extraction, the fibers were briefly rinsed (10 s) in deionized water to remove weakly bound matrix components, then transferred to a desorption plate containing 1 mL of desorption solvent and agitated gently for 30 min. The resulting 1 mL water–acetonitrile mixture (90:10, *v*/*v*) was transferred to clean Eppendorf tubes, and the acetonitrile was evaporated to prevent interference with subsequent analyte separation. The prepared samples were finally analyzed by CE-LIF. Representative electropherograms showing maltose before and after the SPME cleanup procedure to remove excess APTS and 2-picoline from samples are presented in Figure 4.

### 2.3. Method Validation

The analytical method was validated for three monosaccharides and disaccharides commonly present in honey: maltose, mannose, and glucose. Validation parameters included linearity, limits of detection (LOD), limits of quantification (LOQ), precision, and accuracy (see Table 1 and Table 2). Excellent linearity was observed over the tested concentration range of 0.5–10 mM for all analytes, with coefficients of determination (R^2^) exceeding 0.9940. The linear regression models (expressed as peak height versus analyte concentration) confirmed the suitability of the method for quantitative analysis. The calculated LOD and LOQ values were consistent across all validated compounds, at 0.16 mM and 0.5 mM, respectively, indicating satisfactory sensitivity. Precision was assessed as relative standard deviation (RSD) across six replicates at three concentration levels. RSD values ranged from 0.81% to 13.73%, with the highest variability observed at the lowest concentration level (0.5 mM), particularly for mannose and glucose. Nevertheless, the precision was deemed acceptable according to typical analytical performance criteria. Accuracy was evaluated based on recovery studies, with the results ranging from 93.47% to 119.75%, depending on the analyte and concentration level. The highest recovery was recorded for maltose at the lowest spike level (0.5 mM), reflecting both the method’s sensitivity and its robustness across a dynamic concentration range. It should be noted that method validation for fructose could not be completed due to persistent difficulties in its derivatization with APTS. As a ketose, fructose exhibits lower reactivity under the conditions employed for APTS labeling, resulting in inconsistent and unstable signals that precluded reliable quantification. This limitation has been discussed earlier in the context of the method’s development.

A stability study was conducted for glucose, mannose, and maltose stored at room temperature, 4 °C, and −20 °C (data presented in Table S1). Samples were analyzed on days 0, 3, 7, 14, and 21, evaluating peak height (H), peak area, and tailing factor (Tf). Glucose exhibited the lowest stability, with a marked decline in peak area and H at room temperature and 4 °C. At −20 °C, values remained relatively stable up to day 7. Tf also decreased over time at higher temperatures, indicating peak asymmetry likely due to degradation. Mannose remained stable until day 14 across all temperatures but showed significant degradation by day 21 at room temperature. Tf values remained within acceptable limits. Maltose was moderately stable during the first week but showed signs of degradation by day 21, particularly at 4 °C, with Tf increasing slightly over time. Overall, lower temperatures, especially −20 °C, better preserved both analyte stability and peak shape. Glucose was particularly sensitive to storage, highlighting the need to prepare fresh sugar solutions prior to each CZE-LIF analysis. This likely results from the low osmotic pressure of dilute sugar solutions, making them prone to microbial degradation.

#### Dilution Strategy for Samples Exceeding the Validated Linear Range

If the glucose content of a honey sample was found to lie above the validated calibration range of the CZE assay (0.5–10 mM), the aliquot was quantitatively diluted with ultrapure water prior to electrophoretic injection so that the expected concentration fell within the established limits. A ten-fold dilution is generally sufficient; higher factors for dilution may be applied as necessary. To demonstrate that such pre-dilution does not compromise data quality, a dilution-integrity study was performed. Calibration standards at nominal concentrations of 0.5 mM and 10 mM were prepared by diluting stock solutions with water and analyzed in six duplicates. The back-calculated concentrations showed accuracies in the range of 90.4–109.6% and repeatabilities (RSDs) below 12%. These results confirm that the method remains both precise and accurate after dilution. Consequently, any test sample whose analyte level was predicted to exceed 10 mM was diluted—typically 1:10 (*v*/*v*) with deionized water—re-mixed, and re-injected. The measured concentration was then multiplied by the applied dilution factor to yield the true analyte level. The overall validation data, including dilution integrity, comply with the current FDA and ICH bioanalytical guidelines, confirming the suitability of the SPE-CZE procedure for the routine analysis of high-concentration honey samples [29].

### 2.4. Analysis of Real Samples

In this study, four types of nectar honey were analyzed, rapeseed, acacia, phacelia, and dandelion, all sourced from local beekeepers. The selection of samples was based on both their availability and popularity among local consumers, as well as their diverse botanical origins. This approach allowed for the creation of a balanced and representative set of honey samples that reflect the biodiversity and environmental conditions characteristic of the region. The main focus of the analysis was to determine the content of reducing sugars in each sample. This parameter is a key indicator of honey quality and is influenced by factors such as botanical origin, degree of maturity, and storage conditions. The analyses were conducted in accordance with established analytical methods, allowing for an accurate determination of glucose and fructose levels—the primary reducing sugars present in honey.

**Figure 5:**
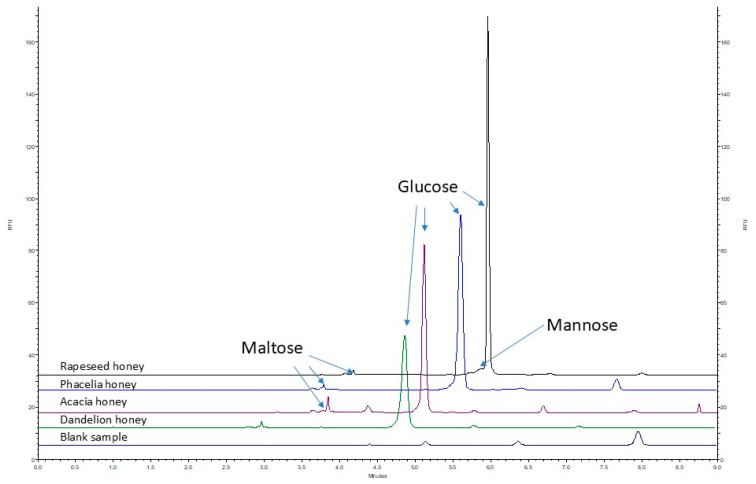
Electropherograms of the analyzed honey samples and blank sample after labeling with APTS and 2-picoline borane.

As presented in Table 3 and Figure 5, glucose concentrations in all the analyzed honey samples exceeded the upper limit of the method’s linear range and were therefore determined using the validated dilution procedure (Section 3.4.1). The applied 10-fold dilution ensured accurate and precise quantification, confirming the method’s suitability for high-glucose matrices, such as phacelia, rapeseed, acacia, and dandelion honey. In contrast, maltose and mannose concentrations remained within the calibration range and were quantified directly without dilution. Mannose was detected only in rapeseed honey, maltose was most abundant in acacia honey, while the highest glucose content was found in dandelion honey. This confirms the method’s applicability for the accurate determination of both major (glucose) and minor (maltose, mannose) sugars in honey samples of various botanical origins.

## 3. Materials and Methods

### 3.1. Chemicals

The analytes glucose (Glu), fructose (Fru), maltose (Mal), and mannose (Man), and the reagents acetone, boric acid, citric acid, sodium tetraborate decahydrate, sodium hydroxide (NaOH), sodium hydroxycarbonate, sodium dodecyl sulphate (SDS), and DMSO were purchased from Sigma-Aldrich (St. Louis, MO, USA). The derivatization agent 8-aminopyrene-1,3,6-trisulfonic acid (APTS) was also purchased from Sigma-Aldrich (St. Louis, MO, USA). The reagents methanol and disodium hydrophosphate were obtained from Merck (Darmstadt, Germany). All chemicals were of analytical grade and were applied without further purification. The Capillary Regenerator Basic Wash (0.1 M NaOH) was purchased from Beckman Coulter (Brea, CA, USA). The deionized (DI) water (18.2 MΩ cm) used in all experiments was obtained from Milli-Q apparatus from Milipore Sigma (Burlington, MA, USA).

### 3.2. Stock Solution of Analyzed Sugars and Honey Samples

Stock solutions of each investigated sugar (individually and as a mixture) were prepared by accurately weighing the appropriate amount of each compound to achieve a concentration of 1 M, followed by dissolution in water. Working solutions with lower concentrations, ranging from 0.5 to 10 mM, were then prepared from the stock solutions. Samples were stored at −20 °C for a maximum of 3 days (glucose, fructose) or 7 days (maltose, mannose).

Four types of honey were analyzed, rapeseed, acacia, phacelia, and dandelion, all sourced from local beekeepers. The selection of samples reflected both their popularity in the local market and botanical diversity, providing a representative overview of honeys characteristic of the region. For the determination of sugars present in the analyzed honey samples, 1 g of each honey was weighed and dissolved in 5 mL of deionized water. The samples were then shaken for 30 s and incubated at room temperature for 15 min. Subsequently, the samples were placed in an ultrasonic bath, centrifuged at 6000 rpm for 5 min, and 2 mL of the supernatant was filtered through a 0.22 µm membrane filter. The prepared honey samples were then subjected to the labeling procedure (see Figure 1).

### 3.3. Instruments

A Beckman P/ACE MDQ system (Fullerton, CA, USA) equipped with a laser-induced fluorescence (LIF) detector (excitation: 488 nm, emission: 520 nm) and a liquid cooling device was used for analysis. All analytes were separated using an uncoated fused-silica capillary (Polymicro Technologies, Phoenix, AZ, USA) with a total length of 50.2 cm (effective length: 40 cm) and an inner diameter of 50 µm. The separation temperature was maintained at 25 ± 0.1 °C by immersing the capillary in a cooling liquid circulating within the cartridge tube. The separation buffer was composed of 20 mM sodium tetraborate decahydrate. The hydrodynamic injection duration was 10 s at 0.5 psi, whereas the analysis time was 12 min. To the capillary, a voltage of 25 kV was applied that generated a current of about 80 µA. A new capillary was conditioned by flushing with 0.1 M NaOH for 10 min, followed by exposure to deionized water for 10 min. Routine daily washing between runs was performed using sequential flushing with 0.1 M NaOH (1 min), deionized water (1 min), and rinse buffer (1 min) under pressure (20 psi) applied at the anodic end of the capillary. Data processing was carried out using 32 Karat system 8.0 software (Beckman).

### 3.4. Sample Preparation Procedures

#### 3.4.1. Sample Derivatization with APTS and 2-Picoline Borane

Three monosaccharides (glucose, fructose, and mannose), a disaccharide (maltose), and their mixtures were subjected to derivatization processes. For this purpose, 10 μL of glucose, fructose, maltose, and mannose solutions or their mixtures with concentrations ranging from 0,5 mM to 10 mM were collected into clean Eppendorf tubes. In the next step, 4 μL 0.1 M APTS in DMSO, 4 µL 0.5 M citric acid aqueous solution, and 4 μL 1.0 M 2-picoline borate solution in the mixture (MeOH:CH_3_COOH, 10:1, *v*/*v*) were added to each sample. The tubes were protected from light and left for 2 h in a thermoblock at 50 °C to perform reductive amination reaction. The derivatized sugar samples were diluted with distilled water to a final volume of 200 μL. Subsequently, a 10 μL aliquot was taken and further diluted 10-fold with DI water prior to CZE-LIF analysis. Then, 50 μL of each prepared sample was collected and subjected to electrophoretic analysis. Samples that were not analyzed immediately after the derivatization process were placed in a freezer and analyzed the following day. The schematic diagram of the procedure for derivation of the tested sugars is shown in Figure 1.

#### 3.4.2. Procedures for the Removal of Excess Reagents from Samples

##### A Liquid–Liquid Extraction (LLE)-Based Approach

To the remaining volume of samples obtained in the derivatization process (190 µL) was added chilled ethyl acetate as an extraction solution (380 µL) in a ratio of 1:2, then the whole mixture was mixed vigorously on a vortex shaker for 30 s. The next step was to centrifuge the samples for 2 min at 10,000 rpm and allow the phases to separate. After phase separation, the upper organic layer containing impurities such as excess unreacted APTS and 2-picoline borane was discarded, and the clear supernatant was collected from the aqueous (lower) part containing the labeled analytes. To remove traces of ethyl acetate from the aqueous part, the samples were evaporated in a thermoblock at 40 °C for 10 min. Then the samples were subjected to separation analysis without additional sample dilution.

##### A Solid-Phase Extraction (SPE)-Based Approach

Similar to the LLE-based procedure, the remaining 190 μL of sample from the derivatization procedure was diluted in a ratio of 1:3 with DI (190 μL sample:570 μL water) and mixed on a vortex shaker for 30 s. The samples were protected from light using aluminum foil. The process of conditioning the SPE C18 columns was carried out by rinsing them successively with 1 mL of acetonitrile, 1 mL of DI, and 1 mL of 100 mM citric acid. Samples of 760 μL were loaded onto the SPE column and passed through the column using a slight vacuum. The columns were rinsed with 1 mL of DI and dried for 5 min in a vacuum. In the next step, elution was carried out with 500 µL of 10% ACN in DI. The obtained samples were heated for 10 min in a thermoblock at 40 °C to remove traces of ACN. The prepared eluate was subjected to CZE-LIF analysis without additional sample dilution.

##### A Solid-Phase Microextraction (SPME)-Based Approach

To the 190 μL of sample obtained after the derivatization procedure (Section 3.4.1.), DI water at a volume of 1000 μL was added, and the sample was mixed on a vortex shaker for 30 s. The samples were protected from light using aluminum foil. Before the SPME procedure, C18 fibers were activated in 1.5 mL of acetonitrile:water (50:50, *v*/*v*) for 30 min, and then they were washed with DI for 10 s. Samples were placed in a 96-well plate and subjected to an extraction process for 30 min using gentle plate mixing. Next, the fibers were immersed in DI for 10 s and then transferred to a 96-well plate containing a desorption solution of 1 mL 10% ACN in DI water, and then a desorption step for 30 min using gentle plate mixing was performed. The desorbent was transferred to clean Eppendorf tubes and traces of ACN were evaporated in a thermoblock at 40 °C for 10 min. The obtained samples were subjected to CZE-LIF analysis without additional sample dilution. SPME fibers were immersed in a fresh portion of desorbent, a carry-over test was performed for 30 min, and the obtained samples were heated at 40 °C for 10 min in a thermoblock before CZE-LIF analysis.

### 3.5. Study of Analyte Stability Under Storage Conditions

Due to observed changes in the peak height and symmetry of analyte signals over time, a stability study of sugars was conducted, taking into account the various storage conditions of the tested solutions. Samples containing the target sugars at a concentration of 10 mM were stored at 20 °C, 4 °C, and –20 °C for a period of 21 days. Aliquots were collected at five time points: days 0, 3, 7, 14, and 21. On each of these days, the respective samples underwent reduction and derivatization according to the procedure described in Section 3.4.1 and illustrated in Figure 1. Subsequently, the samples were analyzed using CZE-LIF.

For each measurement, the peak height and area were evaluated, and the tailing factor (Tf) was calculated to assess peak symmetry. A Tf value in the range of 0.9–1.2 indicates very good peak symmetry, while a value between 1.2 and 1.5 corresponds to acceptable symmetry with slight tailing. Tf values above 1.5 suggest significant tailing, whereas values below 0.9 indicate peak fronting. The tailing factor was calculated using the formula Tf = W_0.05_/2f, where W_0.05_ is the peak width at 5% of its height and 2f is the distance from the front of the peak to its maximum at the same height level.

### 3.6. Validation of the Method

The validation of the CZE-LIF method was carried out in accordance with the international guidelines of the Food Drug Administration (FDA) and the International Conference on the Harmonization of Technical Requirements for the Registration of Pharmaceutical products for human use (ICH). The method was validated for selectivity, linearity, precision, accuracy, and recovery.

### 3.7. Verification of Method Applicability Using Real Honey Samples

To confirm the applicability of the developed method, the contents of glucose, mannose, maltose, and fructose were determined in four types of honey: rapeseed, phacelia, dandelion, and acacia. The honey samples required appropriate preparation prior to analysis. For each sample, 1 g of a given honey type was weighed into Eppendorf tubes, followed by the addition of 5 mL of ultrapure water. The mixture was vortexed for 30 s and then incubated at room temperature for 15 min to ensure the complete dissolution of the honey in water. Subsequently, the sample was subjected to sonication for 10 min.

The resulting aqueous honey solution was centrifuged at 6000 rpm for 5 min. A 2 mL aliquot of the clear supernatant was withdrawn using a sterile syringe and filtered through a 0.22 µm membrane filter (Millex-GP, Millipore, Burlington, MA, USA) into a clean tube. For analysis, 10 µL of the prepared real sample was taken and processed according to the procedure shown in Figure 1 and described in Section 3.4.1.

## 4. Conclusions

This study demonstrates the applicability of 2-picoline borane as a safer and efficient reducing agent for the APTS-labeling of reducing sugars in CZE-LIF analysis. Optimized derivatization conditions enabled the effective labeling of glucose, mannose, and maltose, while fructose required modified protocols due to its lower reactivity. Due to its structural characteristics, fructose required extended incubation to improve derivatization yield, but consistent quantification remained challenging. A novel aspect of this work was the comparative evaluation of LLE, SPE, and SPME cleanup methods, with SPE providing the best balance between derivatization reagent removal and signal intensity. The elaborated method was validated for major monosaccharides and disaccharides, showing excellent linearity (R^2^ > 0.994), satisfactory sensitivity (LOD = 0.16 mM), and acceptable precision and recovery. The validated method showed excellent linearity, sensitivity, and precision for major sugars and was successfully applied to real honey samples, confirming its potential for the routine analysis of complex food matrices.

## Figures and Tables

**Table 1:** Method validation data in terms of linearity, regression equation, LOD, and LOQ.

Analyte	Linear Range [mM]	Regression ^a^	R^2^	LOD [mM]	LOQ [mM]
Maltose	0.5–10	y = 42,274x − 7749.7	0.9940	0.16	0.5
Mannose	0.5–10	y = 173,542x − 71,114	0.9951	0.16	0.5
Glucose	0.5–10	y = 48,804x − 14,430	0.9941	0.16	0.5
Fructose	No data	No data	No data	No data	No data

^a^ *x* value is the concentration of analytes (mM); the *y* value is the peak height (RFU).

**Table 2:** Precision and accuracy validation data (*n* = 6).

Concentration Added [mM]	AVG Concentration Found [mM]	SD	RSD [%]	Accuracy [%]
Maltose				
0.5	0.60	0.06	10.44	119.75
5	4.87	0.07	1.34	97.47
10	10.39	0.08	0.81	103.94
Mannose				
0.5	0.56	0.09	12.97	112.96
5	4.76	0.25	5.32	95.29
10	9.48	0.73	7.68	94.80
Glucose				
0.5	0.55	0.09	13.73	110.13
5	4.67	0.08	1.77	93.47
10	10.44	0.15	1.42	104.41

**Table 3:** The concentration of analyzed sugars in honey samples as their diverse botanical origins (*n* = 3).

Honey Type	Concentration in Honey Samples [mM]		
	Maltose	Mannose	Glucose
Facelia honey	7.94	-	197.4 ^a^
Rapeseed honey	3.84	1.98	121.75 ^a^
Acacia honey	13.4 ^a^	-	133.02 ^a^
Dandelion honey	5.74	-	72.84 ^a^

^a^ The concentration of analytes in real honey samples was determined according the dilution procedure described in Section 3.4.1.

## Data Availability

Data are contained within the article.

## Supplementary Materials

The following supporting information can be downloaded at: https://www.mdpi.com/article/10.3390/ijms26157569/s1.
