# Supplementary material for: Simultaneous Determination of Reducing Sugars in Honey by Capillary Zone Electrophoresis with LIF Detection Using Low-Toxicity 2-Picoline Borane and APTS for Pre-Capillary Derivatization
Source: Int J Mol Sci. 2025 Aug 5;26(15):7569. doi: 10.3390/ijms26157569 (PMC12347702; doi:10.3390/ijms26157569)
Supplement: Supplementary file 1 [file ijms-26-07569-s001.zip › ijms-3764828-supplementary.pdf]

SUPPLEMENTARY MATERIALS

Simultaneous Determination of Reducing Sugars in Honey by Capillary Zone Electrophoresis with LIF Detection Using Low-Toxicity 2-Picoline Borane and APTS for Pre-Capillary Derivatization

Joanna Bulesowska [1], Michał Pieckowski [1], Piotr Kowalski [1], Tomasz Bączek [1,2] and Ilona Olędzka [1,*]

[1] Department of Pharmaceutical Chemistry, Medical University of Gdańsk, 80-416 Gdańsk, Poland

[2] Department of Nursing and Medical Rescue, Institute of Health Sciences, Pomeranian University in Słupsk, 76-200 Słupsk, Polan

Table S1. Stability study of analysed sugars.

**Day**	**Glucose**								
	**Room temperature**			**4ºC**			**-20ºC**		
	H	A	Tf	H	A	Tf	H	A	Tf
0	753100	1498544	**1.15**	-	-	-	-	-	-
3	724307	1427581	0.91	541471	1881241	**1**	467163	1993279	**1.04**
7	692241	1345658	0.71	417043	1828473	0.82	319258	1812103	0.71
14	366507	1234949	0.66	348705	1735923	0.82	237585	1633811	0.70
21	304460	1103656	0.63	166316	981928	0.82	160533	942398	0.74
	**Mannose**								
0	1455503	7456243	0.77	-	-	-	-	-	-
3	1223435	7204500	0.73	1659952	8940237	0.7	2034043	7730309	0.79
7	1196559	6580260	0.81	1642165	8405528	0.69	1947574	5810517	0.69
14	1186728	4640753	0.68	1121878	6222593	0.73	1333104	4927950	0.76
21	152348	214475	0.60	630670	3680348	0.76	736716	2800771	0.72
	**Maltose**								
0	208868	389171	**1.1**	-	-	-	-	-	-
3	205722	353883	**1.1**	581869	1119696	**1.03**	783324	889162	**1.12**
7	197313	321050	**1.04**	543589	1095149	**1.25**	750360	840604	**1**
14	183611	299543	**1.06**	335986	715568	**1.16**	275885	386461	**1.13**
21	-	-	-	198290	621551	0.69	180821	256099	**1.14**

Figures of contents:

Figure S1. Electropherograms of samples containing fructose at a concentration of 10 mM labeled with APTS and 2-picoline borane at 30 °C for different reaction times.

Figure S2. Electropherograms of samples containing fructose at a concentration of 10 mM labeled with APTS and 2-picoline borane at 40 °C for different reaction times.

Figure S3. Electropherograms of samples containing fructose at a concentration of 10 mM labeled with APTS and 2-picoline borane at 50 °C for different reaction times.

Figure S4. The comparison of samples containing 10 mM of fructose after labeling with APTS and 2-picoline for 4h at different temperature.

Figure S5. Comparison of blank samples before and after SPE C18

Figure S6. The electropherograms clearly show significantly reduced signals corresponding to excess APTS in the sample subjected to the SPE C18 procedure vs sample without purification SPE.

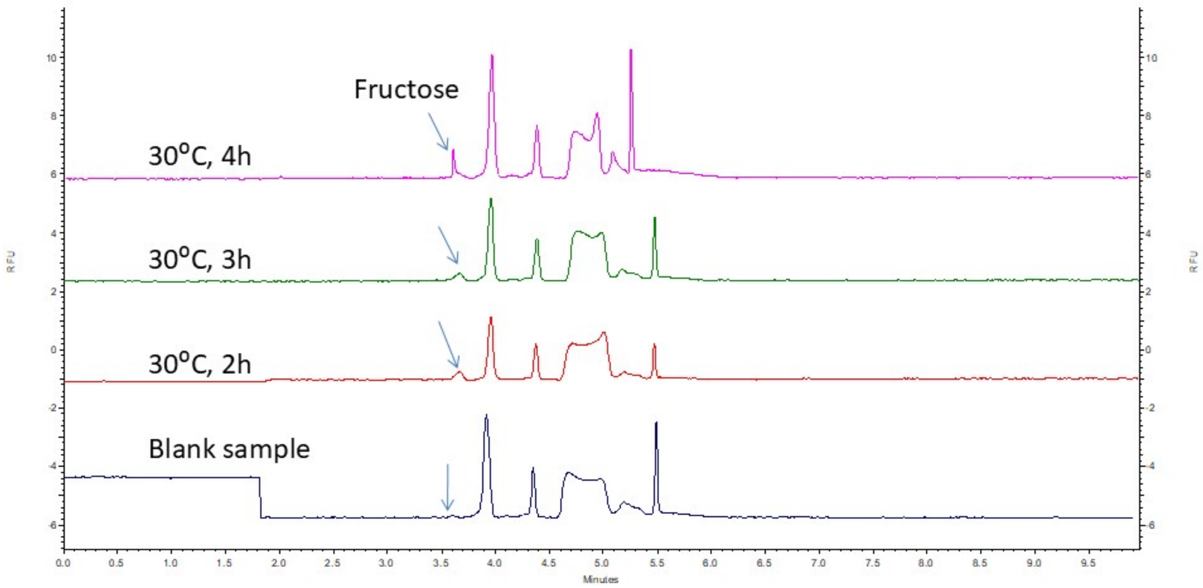

Figure S1. Electropherograms of samples containing fructose at a concentration of 10 mM labeled with APTS and 2-picoline borane at 30 °C for different reaction times.

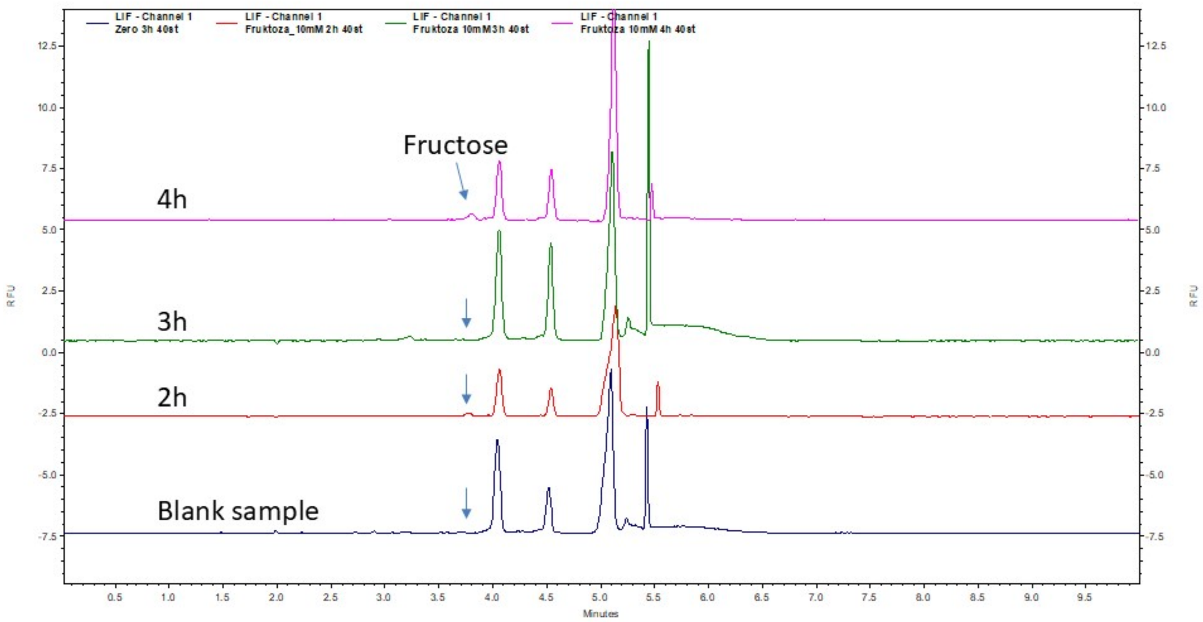

Figure S2. Electropherograms of samples containing fructose at a concentration of 10 mM labeled with APTS and 2-picoline borane at 40 °C for different reaction times.

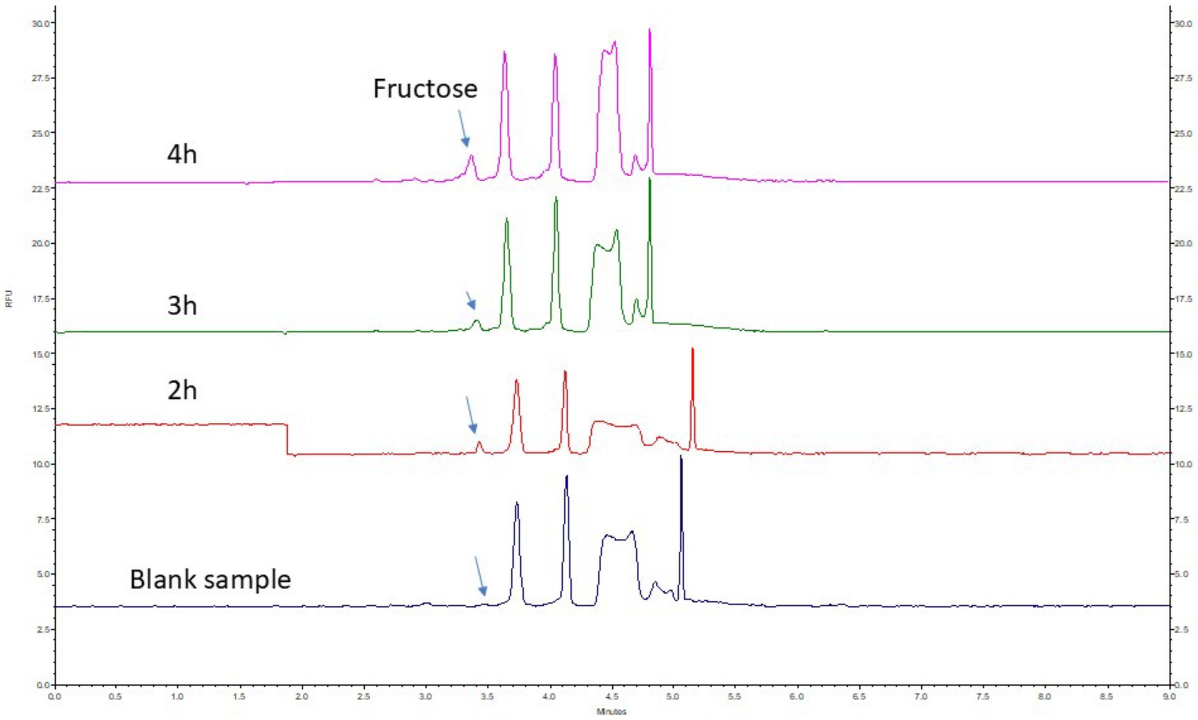

Figure S3. Electropherograms of samples containing fructose at a concentration of 10 mM labeled with APTS and 2-picoline borane at 50 °C for different reaction times.

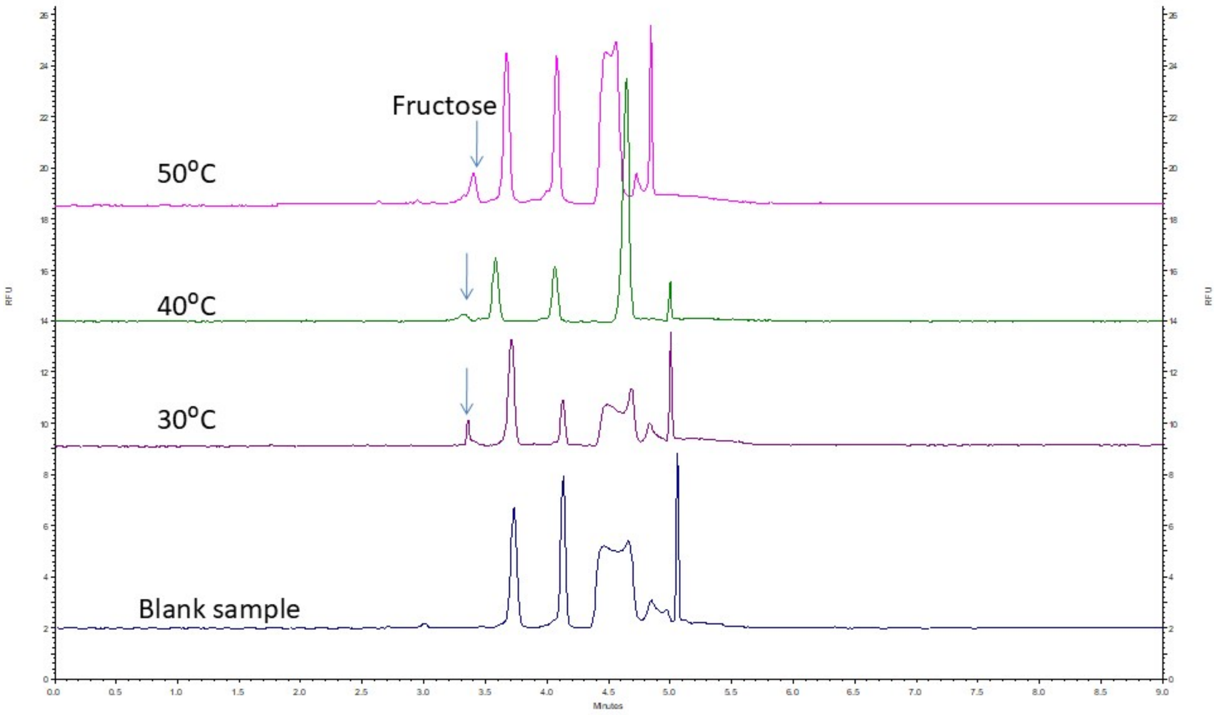

Figure S4. The comparison of samples containing 10 mM of fructose after labeling with APTS and 2-picoline for 4h at different temperature.

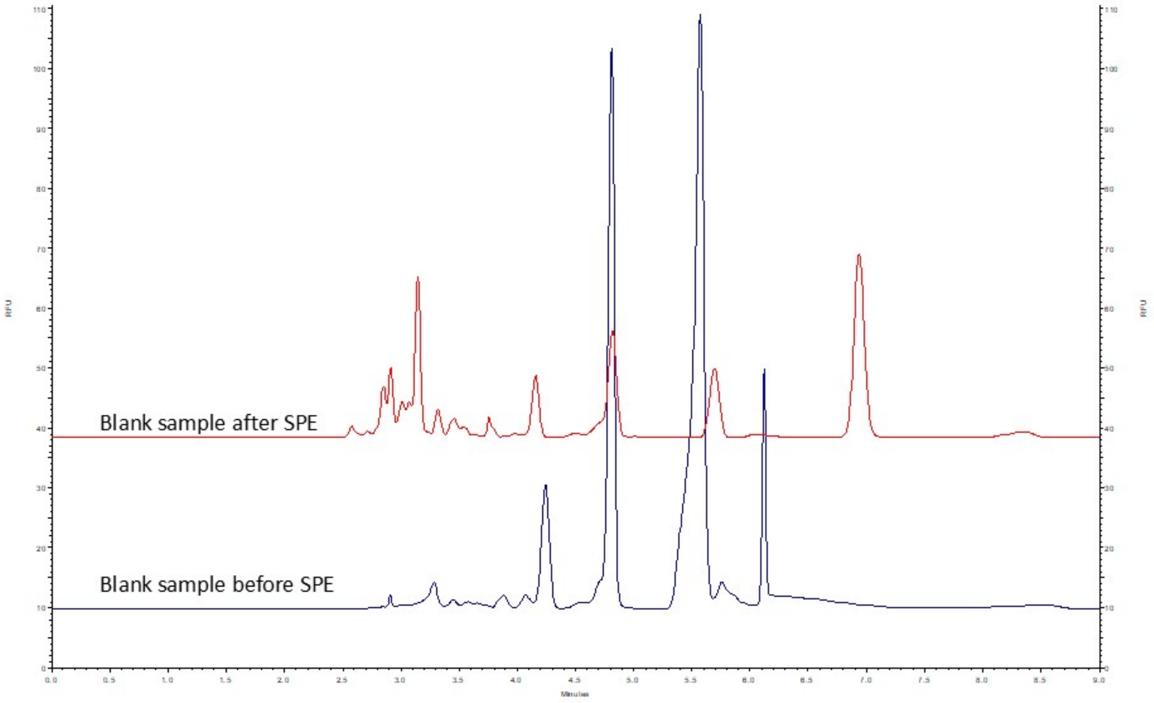

Figure S5. Comparison of blank samples before and after SPE C18

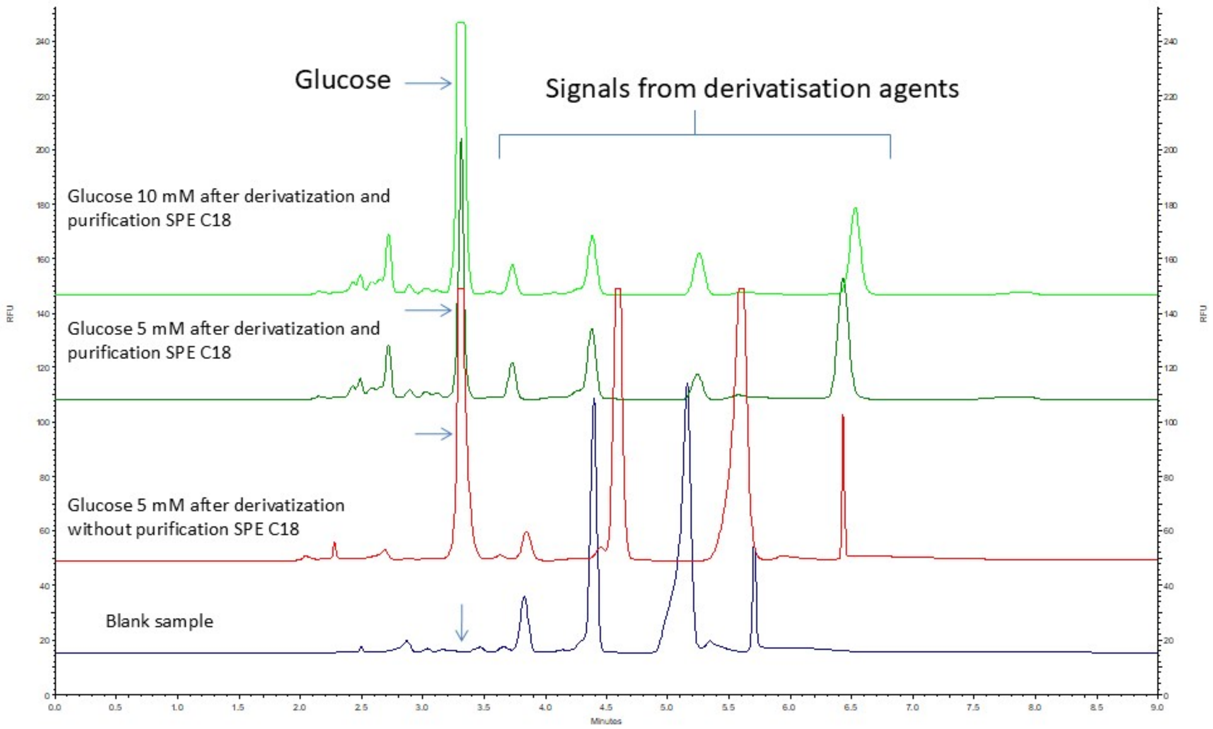

Figure S6. The electropherograms clearly show significantly reduced signals corresponding to excess APTS in the sample subjected to the SPE C18 procedure vs. sample without purification SPE.
